# Should children under 5 and those with constipation be overlooked from ARFID research? An open response to “Subtypes of avoidant/restrictive food intake disorder (ARFID) in children and adolescents: a latent class analysis”

**DOI:** 10.1016/j.eclinm.2024.102667

**Published:** 2024-06-03

**Authors:** Celine Hall, Victoria Illingworth, Annemarie Sims, Gabriel Whitlingum, Jo Cryer

**Affiliations:** Complex Feeding Team, Department of Paediatric Neurosciences, Evelina London Children’s Hospital, Guy’s & St Thomas’ NHS Foundation Trust, London, UK

The recently published study “Subtypes of avoidant/restrictive food intake disorder (ARFID) in children and adolescents: a latent class analysis” purports to explore the likely subgroups and incidence of ARFID, however, its exclusion of children with constipation and those under 5 raises significant concern. By overlooking this crucial subset of affected children, the study underestimates the true caseload. It also overlooks a prevalent aspect of ARFID but fails to acknowledge that constipation is a common symptom of ARFID, resulting from a predominantly carbohydrate-based diet. Moreover, failing to include this subset of children may hinder effective diagnosis and treatment of ARFID.

We note that, in the survey group, children and young people (cyp) < 5 years were excluded although there is no minimum age for ARFID diagnosis according to DSM and ICD criteria. We also note that many cyp with allergy, constipation, and other conditions were likely to have been excluded, although in our experience these commonly co-occur as factors in cyp with ARFID but are not the ongoing factor in the pattern of disordered eating.

In our paper that reviewed a caseload of over 500 children seen in our Complex Feeding Clinic–Avoidant/restrictive food intake disorder and autism spectrum disorder: Clinical implications for assessment and management (Farag et al., 2022), we diagnosed ARFID in just under 10% of our caseload in the 0–3 category and just over 73% in the 4-9-year age group, with a mean age overall of 6 years 6 months. We found younger children with autism spectrum disorder were more likely to have significant nutritional deficiency so it is vital that these children are not overlooked.

## Contributors

Celine Hall: project administration; writing—original draft and editing.

Dr Victoria Illingworth: writing–original draft.

Annemarie Sims: writing–original draft.

Dr Gabriel Whitlingum: writing–original draft.

Dr Jo Cryer: writing–original draft, supervision.

## Declaration of interests

Jo Cryer and Annemarie Sims are trustees for ARFID Awareness UK.

